# Improving family functioning and wellbeing among families of children with disabilities in Rwanda: an application of the SAFE child protection model

**DOI:** 10.3389/fpsyt.2026.1906171

**Published:** 2026-09-14

**Authors:** Alain Ahishakiye, Hannah Gilbert, Leila Dal Santo, Ann Miller, M. C. Smith Fawzi, Cat Kirk, Candace J. Black, Wijnand Van Den Boom, Mukeshimana Verena, Roberto Baiocco, Sarah K. G. Jensen, Kim Wilson, Vincent Sezibera, Theresa S. Betancourt

**Affiliations:** 1 Center for Mental Health, University of Rwanda, Kigali, Rwanda; 2 Harvard Medical School, Global Health Department, Harvard University, Boston, MA, United States; 3 Research Program on Children and Adversity, Boston, MA, United States; 4 Sapienza University of Rome, Department of Developmental and Social Psychology, Roma, Italy; 5 Independent Researcher, Pediatric Developmental Clinic, Partners In Health/Inshuti Mu Buzima, Rwinkwavu, Rwanda; 6 Boston Children's Hospital, Boston, MA, United States; 7 Harvard Medical School, Boston, MA, United States; 8 Ndera Neuropsychiatric Teaching Hospital, Kigali, Rwanda

**Keywords:** caregivers of children with disabilities, family functioning, family wellbeing, father engagement, parenting program, SAFE model, young adult

## Abstract

**Background:**

Families of children with disabilities in low-resource settings face heightened challenges to family functioning and wellbeing. Improving a child and caregiver’s environment in these settings requires a broader understanding of the family ecosystem. This study aims to investigate factors that should be considered to enhance family functioning and inform family-centered interventions.

**Methodology:**

We conducted 34 in-depth interviews with 30 caregivers of children with disabilities aged 6–8 years enrolled in a larger cluster randomized trial of the Sugira Muryango parenting intervention. Additionally, six focus group discussions were conducted with 36 community workers, and 6 Early Childhood Developmental (ECD) advocates participants. Guided by the SAFE Model for child protection, content analysis was used to understand threats to child protection and caregivers’ well-being among caregivers of children with disabilities enrolled in Sugira Muryango.

**Results:**

Consistent with the domains outlined in the SAFE Model, our analysis revealed the following threats to child safety: 1) Safety/Freedom from harm, including father’s mental health and substance abuse, abandonment and stigma related to a child’s disability; 2) Access to basic physiological needs and healthcare, including lack of basic necessities (food, utilities and housing), additional needs related to caring for a child with disabilities, caregiver mental health needs, limited access to health insurance and mother illness; 3) Family and connection to others, including the single-parent dilemma, lack of support from family and community, and father abandonment; and 4) Education and economic security, including additional expenses due to the child’s disability and financial stress related to health care costs.

**Conclusion:**

Improving family functioning and wellbeing among caregivers of children with disabilities in limited resources requires a deep understanding of socioecological forces operating on the family system. These findings provide further evidence of how the threats to child safety highlighted by the SAFE Model can inform how researchers, and policy makers design and implement family-centered interventions tailored to children with disabilities in low-resource settings.

## 1 Introduction

The UN Convention on the Rights of the Child lays out the importance of safety, security and a health promoting environment for children to reach their full developmental potential (1–3). Families raising children with disabilities, particularly those in low-resource settings like Rwanda, face overwhelming challenges in securing these conditions for their children due to psychological, financial and social constraints (4).

In addition to the mental health risks associated with trauma exposure and loss of family experienced during the genocide in Rwanda, the cumulative emotional and physical stress related to raising a child with disabilities as a single parent can compromise caregiver’s mental health and child well-being (5). Despite this psychological toll, few mental health professionals (particularly in rural areas) exist to support the mental health needs of caregivers of children with disabilities (6). Moreover, access to healthcare–whether for themselves or their children–remains a real challenge for caregivers who rely on daily wages to pay for their basic needs (e.g., food, clothes, and hygiene items).

Beyond the precarity in securing basic household, healthcare, and educational needs, families of children with disabilities are especially vulnerable to discrimination and social isolation, most notably in rural areas (7). Indeed, research has demonstrated that in such environments, mothers and children with disabilities are subject to blame from the father and the community (8). Father abandonment among families with a child with disabilities is well-documented, resulting in increased social isolation, a reduction in the family’s social network, and more limited access to social benefits. Such stigma has even resulted in children with disabilities being moved from their families and placed in an institution or a foster care family–this despite global recognition that institutionalizing children can be unsafe and not conducive to the development of the child (9, 10).

The Rwandan government has demonstrated strong leadership and a commitment to promoting the rights of children living with disabilities, implementing progressive reforms that prioritize the reintegration of children with disabilities back to their biological families (when possible) and advancing family centered interventions that aim to improve caregiver knowledge and capacity to provide appropriate care for children with disabilities (11).

The recent universal health insurance update is also expected to explicitly consider families of children with disabilities living in limited resource situations who were previously overlooked (12) as part of the lowest income bracket, therefore making these families eligible for a full fee waiver (13).

On the research front, researchers in Rwanda have applied a holistic approach to inform policymakers and researchers on how best to improve the wellbeing of families living in adversity (14). However, there are still gaps in our understanding of the factors across the ecological framework that influence the wellbeing of families of children with a disability. Such a holistic approach and deeper analysis of the threat to the rights of children with disabilities and their caregivers is necessary to inform policymakers’ decisions and the design of parenting interventions to alleviate the burden of negative social forces and improve family functioning and caregiver-child wellbeing. This study aims to examine the socio- ecological factors that are involved in the context of a parenting program to inform its adaptation to the specific needs of families with disabilities and to advise policymakers regarding how best to regarding advance child protection to support parenting programs for this subpopulation for families of children with disabilities.

## 2 The SAFE model

The SAFE model is a socio-ecological model used to identify key threats and opportunities related to advancing child protection and serves as the framework underpinning this study. The model is especially useful for examining various dimensions of child protection in the context of adversity and has previously been used by researchers in Rwanda (15), including among households affected by HIV/AIDS (16) (**Table 1**).

**Table 1:** SAFE model themes.

S	SAFETY/FREEDOM from harm
A	ACCESS TO PHYSIOLOGICAL NEED AND HEALTHCARE
F	FAMILY AND CONNECTION TO OTHERS
E	EDUCATION/LIVELIHOODS AND SENSE OF FUTURE

The SAFE framework includes: 1) Safety and freedom from harm: child protection implies a safer family environment that mitigate child development threats. Child protection is interlaced throughout the system, with household safety promotion, violence-prevention mechanisms, and community child protection structures grounded in Rwanda’s Child Protection Policy and global evidence on safe caregiving environments (17). 2) Access to health care and physiological needs involve health services from the first protective cradle, where perinatal care, immunization, growth monitoring, and the early detection of developmental concerns safeguard children’s survival and developmental potential (18). Parallel to this, nutritional care nurtures the body and brain, through optimal infant and young child feeding (IYCF), micronutrient supplementation, and close monitoring of growth trajectories (19). 3) Family and connection to others: Responsive caregiving—another pillar of nurturing care—finds expression in parenting education, caregiver support initiatives, and home-visiting programs, strengthening attuned, emotionally present caregiving environments (20). Meanwhile, family connection with others including neighbors provide an opportunity for their children to interact and allow play-based learning (21) along with community ECD centers. The in and out home stimulation call to anchor early childhood systems in enriching, developmentally transformative experiences.4) Education/Livelihoods and a sense of future refer to the ability to provide for the family, including access to income generating activities, to school supplies for the education of their children, and skills development (16). Meeting these needs requires inclusive planning, which is the first safeguard, ensuring that no one is left behind in policy or practice.

Previous scholars have leveraged the Community-Based Rehabilitation (CBR) model, an internationally recognized model for strengthening access to rehabilitation and participation in the building of society in low-resource contexts to understand the challenges caregivers face was (22). For example, a scoping review (23) conducted in 2024 using the CBR framework explored its effectiveness to improve the livelihood of caregivers of children with disabilities. However, this model is not sufficient to our understanding of how certain domains, specifically those outlined by the SAFE model, impact family functioning and wellbeing. I Specifically, the SAFE model recognizes that alongside improving livelihood, ensuring a safer environment for childhood development implies improving family functioning and well-being.

## 3 Methodology

### 3.1 Study purpose and setting

The purpose of this qualitative study is to understand factors that can be mitigated to improve family functioning and foster child protection among families of children with disabilities. This study was conducted between September and October of 2023 in three districts of rural Rwanda: Ngoma, Rubavu, and Nyanza. Caregivers were recruited for participation in this study if they had received the Sugira Muryango intervention between 2018 and 2019 and had a child with a disability (scored positive using the 7 items adapted from the UNICEF 10-item screener, Multiple Indicator Cluster Survey version) (24). Note that families eligible for participation in Sugira Muryango were classified by the Rwandan government as living in extreme poverty using rank in the Ubudehe classification (25).

### 3.2 Sugira Muryango intervention and study population

The Family Strengthening Intervention (FSI), also known as “Sugira Muryango” in Kinyarwanda, is a home-visiting intervention that uses psychoeducation and active coaching of caregivers to promote responsive caregiving, nutrition, hygiene, and nonviolent interactions among household members. Each home visit includes a 15-minute active play session, in which caregivers receive live feedback on parent-child interactions to support responsive care following the UNICEF and WHO Care for Child Development package and WHO Nurturing Care Framework. Flexible scheduling and messaging during the visits encouraged this engagement of male caregivers in play and nurturing care. Coaches also help families navigate formal resources (e.g., government programs to promote child health and nutrition, including supplemental nutrition for malnourished children) and informal support from neighbors, extended family, and community members. The effectiveness of the program was assessed as part of a cluster randomized trial conducted between 2018 and 2019. Details of this trial, in which families in three districts (Nyanza, Ngoma, and Rubavu) participating in the government’s public works program were randomized to treatment or control, are reported elsewhere (25). The Sugira Muryango intervention was implemented between May and September of 2018 (the intervention was staggered to ensure it started within two weeks of baseline data collection) and follow-up data were collected in 2019.

### 3.3 Participant sampling and recruitment

The study population was limited to participants in a larger study assessing the impact of the Sugira Muyango intervention; details of the study can be found elsewhere. (5, 25). Purposeful sampling was used to select caregivers of children with disabilities with low- and high-risk for poor mental health outcomes, defined as anxiety or depression on the Hopkins Symptom Checklist (HSCL-25). An ordered list of the highest and lowest predicted probabilities for poor mental health was generated, and participants were approached for qualitative interviews (**Table 2**). A total of 34 in-depth interviews were conducted with 30 caregivers (10 from each of 3 districts) who met this low- and high-risk profile. The children were enrolled in 2018 aged 12–36 months and screened positive for some form of developmental delay identified using the 7 items adapted from the UNICEF 10-item screener, Multiple Indicator Cluster Survey version. The children were aged 6–8 in 2023 at the moment of the study.

**Table 2:** Qualitative data collection: characteristics of participants.

Participants	In-depth interview (N = 34)			Focus group (N = 6)		
	Rubavu	Nyanza	Ngoma	Rubavu(N = 2)	Nyanza(N = 2)	Ngoma(N = 2)
Marital Status						
Single parents	**8**	**8**	**8**			
Married/Cohabitating	**2**	**2**	**2**			
Poor mental health risk (N = 30)						
Low (N = 15)	**5**	**5**	**5**			
High (N = 15)	**5**	**5**	**5**			
CHW				**8**	**8**	**8**
Lay workers				**4**	**4**	**4**
ECD leader				**1**	**1**	**1**
Social Protection leader				**1**	**1**	**1**
Health care Providers PDC Kirehe and Rwinkwavu						
Pediatrician	**`1**					
Nurses	**2**					
Social worker	**1**					

Bold values means in depth interviews N =34 is the number of total participants and for the focus group the N= 6 is the total number of focus group with 42 participants as the total number for all the 6 focus groups.

Additionally, we interviewed four health providers, including one pediatrician, two nurses, and one social worker employed at the Pediatric Developmental Clinic. These health care providers had a direct, close interaction with caregivers of children with disabilities at the clinic. They were purposefully targeted because we aimed to understand the everyday experiences, stresses and support systems involved in taking care of children with disabilities.

Finally, we conducted six focus group discussions (two in each of the three districts) with lay workers (n=24), community health workers (n=12) (CHW), and early childhood development (ECD) advocates (n=6).

The participants from the focus group were purposefully selected because they were daily engaging with families of children with disabilities, providing support and care. They had valuable insights on the daily living of the caregivers and their children in the community. They were standing up advocating for these families. The lay workers were the interventionists who implemented the CRT intervention. The CHW and ECD advocates were working daily with families of children with disabilities. They were also advocating for these families. Therefore, they had firsthand knowledge and deep experience working with the caregivers of children with disabilities.

Each focus group was constituted by 6–7 participants to ensure saturation was reached (26).

### 3.4 Reflexivity

The researchers were mindful of how their personal experience may shape the way they were reading other people’s experiences. The researchers, being from a similar cultural context, helped to interpret the findings closer to the participants’ reality. The researchers were also mindful about their gender identities, beliefs and societal privileges knowing that these personal factors may have impacted how they collected and analyzed the results. To try to reduce that the researchers were both genders and met over the course of the data analysis to discuss potential sources of bias.

### 3.5 Coder agreement

Researchers met every week for a period of 2 months to discuss to go over any discrepancies and resolve any differences. The researchers were engaged in a rigorous, iterative collaborative analysis. The researchers reviewed systematically the emerging codes, resolving any coding discrepancy through a consensus and refined the final themes to be considered.

### 3.6 Key procedures

Each caregiver participated in a single qualitative semi-structured interview of approximately 60 to 90 minutes. We conducted semi-structured interviews after obtaining consent in a private place of the participant’s choosing, predominately a private space within their homes. Interviews were conducted by the first author and his assistant (also co-author on this paper) in Kinyarwanda, the local language, and were recorded with participants’ permission. We used a semi-structured interview guide covering key concerns about children with disabilities, caregivers’ well-being and mental health, and general factors that shape their livelihood.

Healthcare providers were reached by phone to schedule an appointment. The researcher presented each provider with a paper-based consent form explaining the purpose of the study and requesting the pediatrician’s consent to participate. Interviews with the nurses and social worker were conducted in Kinyarwanda while the interview with the pediatrician was conducted in both English and Kinyarwanda. All interviews with healthcare providers were approximately 60 to 90 minutes in duration. All semi-structured interviews with providers were recorded with permission. The semi-structured interview guide for the pediatrician addressed the following topics: experiences caring for children with disabilities, key concerns about children with disabilities, the effects of childhood disability on caregivers and families, and social and economic considerations related to childhood disability.

Meanwhile, nurses and the social worker were queried about key concerns about children with disabilities, caregivers’ well-being and mental health, and general structural concerns.

The lay workers, CHWs, and ECD advocates were identified with the help of the Sugira Muryango program manager and field supervisor and contacted by phone to participate in the focus group discussions. Paper-based consent forms were presented to all FGD participants, and upon obtaining consent, FGDs were conducted in a private space. Each FGD lasted between 60 and 90 minutes, was conducted in Kinyarwanda, and was recorded with participants’ permission. The semi-structured interview guide used for FGDs covered the following topics: the delivery process of Sugira Muryango, key challenges encountered by families of children with disabilities, and key observations from families of children with disabilities.

### 3.7 Analysis

Qualitative data was analyzed using inductive content analysis (27). Following a complete review of the data, a subset of transcripts was open coded. Passages relevant to child protection and caregivers’ well-being were identified and labelled with descriptive phrases, which were then transformed into a draft codebook. The codebook was piloted and revised, and the final codebook was used to code the entire dataset with the assistance of Dedoose qualitative data management software version 9.0.107. The coded data was analyzed inductively to generate a series of draft themes, which were then iteratively refined into a final set of themes related to the SAFE model.

### 3.8 Ethical approval

Ethical approvals were obtained from Harvard IRB and the Rwanda National Ethics Committee.

## 4 Results

Our Study population was harmoniously distributed in the three districts. In each district we conducted in depth interviews with 8 single parents and 2 married couples. In each district we had 5 caregivers at high risk of poor mental health and 5 at low risk of poor mental health outcomes. We conducted, in total, 6 focus group discussions comprising 7 participants in each session and 2 focus group in each district.

The participants from the focus group and in-depth intervention reported information which were then analyzed and the themes emerged in line with the SAFE Model.

The themes of this framework are interrelated and should be view integrated in a synergic way. Tackling them separately would not be as illuminating as beneficial compared to treating them in a an integrated way to determine holistic solutions to improve that improves family functioning.

As a matter of fact, different member of the family are interlinked. The absence or sickness of one of the primary family figures (such as the father) negatively impacts the family unity, cohesion and functioning. From the narrative of the caregiver’s experience emerged themes in line with the SAFE Model describing a cascade of hardships that leads to the family destabilization and vulnerability. Caregiver and their children with disabilities are left insecure and in survival mode.

### 4.1 Safety/freedom from harm

This theme describes the need for protection from physical, emotional, and psychological harm. In the context of our study, several factors highlight threats to safety:

#### 4.1.1 Father’s mental health and substance abuse

It was reported that fathers were struggling with mental health conditions, alcohol, and substance abuse. Father’s mental health led to neglect and, at times, violence, putting both the mother and child at risk of living in an unsafe home environment.

“It was an extremely challenging situation, dealing with my own health condition, my children’s problems, family conflicts, and their father’s mental health issues. There were instances when he forced us, including the little kids and me while pregnant, to spend the night outside the house. It was a complex array of problems in our lives**.” Rubavu (Single Mother)**

#### 4.1.2 Abandonment and stigmatization related to a child’s disability

Father’s abandonment after the delivery of a child with a disability was frequent in our interviewees. Participants noted that healthy children were readily identified as belonging to their father, while disabled children were identified as belonging to their mother. When fathers abandon the family, mothers often face heightened emotional stress and increased vulnerability to harm, particularly when left alone to care for a disabled child.

“During our visits, especially in one family with a single parent, the father left after the birth of a child with disabilities, deeming the child “useless.” The mother bears the burden alone, facing the transportation and caring challenges.” **Rubavu_Community healthcare worker.**

Caregivers deplored the irresponsibility of the fathers who were still around but denied any financial and emotional support to their wives leading to emotional distress and family disconnection.

There are numerous obstacles in their way as they grow up, and it makes me blame their father, who is still living. I am unhappy and furious to watch him strolling around without providing for the children. **Ngoma_ Single Mother**

### 4.2 Access to basic physiological and healthcare needs

Participants spoke in stark terms about their physiological needs, highlighting how families struggled to access clean water, sufficient food, and adequate shelter. They struggled to connect their children to basic healthcare, including immunization and wellness visits, as well as more specialized care including early detection of developmental delays and specialized mental health support.

#### 4.2.1 Lack of basic physiological necessities

Participants mentioned how not being able to provide for their children’s basic needs disturbed their worldview, increasing mental trauma, and lowering their self-esteem.

These thoughts arise when my children inquire about foods, and my inability to provide for them angers me, distorting my perception of the world and making me feel ashamed for still being here. The absence of goats and agricultural land adds to my distress, causing difficulty in falling asleep at night. **Ngoma_Single Mother.**

#### 4.2.2 Healthcare and specialized needs

Families faced significant financial strain due to the medical and caregiving demands of children with disabilities, requiring significant out-of- pocket costs.

In our village, some mothers care for impaired children, but they face obstacles such as financial difficulties that make it difficult for the children to attend school and for the physically disabled child to obtain crutches. Some in my community are mentally challenged; aside from the medication we get from Nyanza health facilities, they receive no further assistance. Family financial incapacity is thus an issue. – **FGD_CHW_NYANZA.**

#### 4.2.3 Limited access to health insurance

Participants noted that, while access to health insurance is mandatory in Rwanda, a recent change in eligibility criteria for public health insurance meant that some families were unable to qualify.

“Since they updated the ubudehe classifications, we were moved out of the first group, and we have yet to seek medical attention since I cannot afford healthcare insurance. Moreover, there have been instances when my child experienced a recurrence of his health condition.” **Ngoma_ Single Mother.**

Without this vital public support, families struggled to pay for their children’s services, with some families selling their belongings or incurring debt in order to cover healthcare expenses.

Financially, we had to make significant sacrifices. We sold our livestock and even our land to afford medical assistance for our child. Even though our resources ran out before we could fully cover the costs, we did everything to ensure our child received the care she needed … It wasn’t easy at all to the point that we sold our lands and our life stocks so that we could afford medical assistance for our child. **Nyanza_ Married Couple.**

#### 4.2.4 Mother illness

Parental illness compounded the challenges facing these families. Households headed by single mothers were particularly vulnerable, because when these mothers became sick they were unable to care for their children or the needs of the household. Sick parents reported that food insecurity exacerbated their illness and made it difficult to follow medical advice while also trying to keep feeding their children with developmental delay. This overwhelming situation impacted their mental health, increasing their anger and irritability, destabilizing their mental health. These caregivers found themselves relying on daily medical and food support from their neighbors.

Accessing recommended diet and prescribed medication improved their medical condition and stabilized them. Food security and access to healthcare brought reassurance and hope for a safer recovery.

After being diagnosed with cervical cancer, I received advice to refrain from certain activities. I couldn’t lift the child, fetch water, or cook. They instructed me to spend 20 minutes sitting and the rest of the time lying down in a specific position. With young children to care for, obtaining the recommended diet, which included fish and bananas, posed a challenge. My kids also struggled to find suitable food. In these circumstances, I reached out to my neighbors, explaining my need for food to support my medication and, if possible, asking for assistance with my children’s needs. This became a part of my daily routine during that period. **Ngoma_Caregiver**

“ ….becoming angry and irritated occurred, and by this time, I have heart disease, HYPERTENSION, which is improving as I am on therapy. I have reservations about that because I’ve been dealing with hypertension for the past five years. However, I acknowledge that this issue could potentially exacerbate its severity. Presently, I am in good health and stability, and my doctor has recognized the improvement in my condition. When my doctor inquired about how I maintain stability in managing my illness, I explained that I adhere to a recommended diet, take medications as prescribed, and follow all their advice diligently. I am genuinely reassured about the prognosis of my illness, and I firmly believe that with continued adherence to the prescribed regimen, it will eventually be cured once and for all.” **Rubavu, Caregiver**

### 4.3 Family and connection to others

This theme emphasizes the importance of family support, caregiving, and a sense of belonging to the community while ensuring children’s well-being. A child with developmental delays, growth and emotional stability depend on family well-functioning to ensure safe attachment. Family functioning used to rely on the support of extended family and connection to others. However, caregivers reported a dynamic change as the extended family and neighbors are dealing with their own challenges. A stable community provides a critically supportive view of the world and a sense of belonging that are crucial elements of child’s security.

“Love seems to have vanished from this world. In the past, we could rely on support from extended family members and husbands after giving birth. Nowadays, it feels like you’re on your own. Even our neighbors, who are struggling like us, can’t offer much assistance.” **Nyanza_ Single Mother.**

#### 4.3.1 Single-parent dilemma

Single mothers struggle with caregiving responsibilities while trying to secure an income, often feeling trapped by the dual roles they must fulfill.

I don’t even have a place to stay. Even the father of my child rejects us and refuses to provide support. I often contemplate seeking employment in Kigali to purchase land. Still, I can’t leave because my mother is elderly and unable to care for the children or manage finances when I’m away. We live day to day. …Sometimes, I wonder if I’m meant to be alive, comparing myself to others. When I talk to the father about school expenses, he denies any responsibility, claiming the child isn’t his. **Nyanza_ Single Mother.**

#### 4.3.2 Lack of support from family and community

Some participants reported feeling isolated and abandoned by their communities, which exacerbates their vulnerability and stress. Participants expressed their concern about the government’s goals related to reducing poverty. They felt left behind by their local leaders. The government of Rwanda put in place economic advantage opportunities to reduce poverty in rural Rwanda through strong strategies including defining those eligible for financial support (household wealth ranks categories), providing cows for poor families, community works on Saturdays and community meetings. However, navigating government bureaucracy to get access to these opportunities is not easy for the most vulnerable families with limited resources and suffering community stigmatization and discrimination in rural Rwanda.

“In this community, people manage their affairs, and it appears improbable that anyone would step in to address your specific situation. There exists a discrepancy, with some individuals wasting food while others endure hunger and a noticeable absence of communal oversight. It might be surprising to find that certain individuals go to bed without a meal, despite leaders actively participating in Imihigo, where their goal is to alleviate poverty and showcase the eradication of poverty in the area. -**Ngoma_ Single Mother**

**“**From what we’ve observed, community meetings take place every Tuesday. During these gatherings, the government exclusively addresses its action plans, focusing on aspects like health insurance, crime prevention, and enhancing security. Surprisingly, the crucial topic of disability awareness is never brought up, despite the potential benefits it could offer to many individuals.” **FGD_ Rubavu**

Caregivers reported that the local non-government organization helped to navigate bureaucracy and build family unity.

“I had the experience; I used to go home and live there for a long time, and I returned here to this home with the help of members of Sugira Muryango after they taught us. And they persuaded me that I needed to come home to my husband, and I agreed … Yes, they assisted us in reuniting our family, living together, and providing a proper education for our children, for which we are grateful … We’ve seen that the teachings we gained from this program formed the foundation for everything in this situation, which also taught us that we could live as a happy, cooperative family.” **Rubavu Married couple**

### 4.4 Education and economic security

This theme focuses on the right to education and the economic security of families, which ensures stable environments for children. While Rwanda is shifting from a lower income country to a middle-income country, ensuring equitable growth among the whole population is challenging. The government of Rwanda has made a significant impact in reducing both acute poverty and unequal distribution of wealth. But still, families of children with disabilities need more attention to maintain a stable society in which children with disabilities and their family may grow safely.

#### 4.4.1 Child’s disability entails additional expenses

The need for continuous caregiving limits mothers’ ability to work, significantly affecting the family’s financial stability. Caregivers of children with disabilities reported a huge burden of carrying their children, making it difficult for mothers to engage in income-generating activities impacting their wellbeing.

The caregiving experience for a child with profound disabilities can be incredibly demanding and all-consuming. Such a child may require constant attention, whether it’s for sleeping or staying close, making it challenging for the mother to return to work … As the child’s disability becomes more complex, the impact on the caregiver’s stress levels intensifies. **HCW_Ped.**

Caregivers of children with disabilities reported increased prices of the school supplies related to global inflation. In fact, they said that they are not able to cover the education of their children. A married caregiver said that she cannot count on the support of her husband who is consumed by alcoholism. On the other hand, the support from the government vanished related to the loss of eligibility due to the new changes in social welfare categorization.

I have a husband who struggles with alcoholism. Unfortunately, I cannot afford schoolbooks for our children or pay for their health insurance since the prices have increased, and we must cover the costs because we no longer support the ubudehe categories. **Nyanza_ Married Couple**

#### 4.4.2 Financial stress related to health care cost

Families of children with disabilities were spending money on basic necessities and healthcare. Most caregivers reported that out-of-pocket healthcare payments cause severe financial stress, compounding economic difficulties. Without government support, caregivers reported not being able to pay health care expenses.

Financial assistance is crucial for mothers with disabled children, especially for medical care. Lack of access to healthcare due to poverty exacerbates the challenges faced by these families - **FGD_CHW_NYANZA**

## 5 Discussion

Caregivers and stakeholders involved in the care of children with disabilities provided their input related to factors to consider in improving the family functioning among families of children with disabilities in rural Rwanda. Findings from the in-depth interviews and focus group discussion in this study aligned with the UNICEF protective environment concept including the interracted four themes outlined in the SAFE framework: safe and freedom from harm, access to basic physiological needs, healthcare, family and connection with others, education, and economic needs.

With respect to freedom from harm and safety, caregivers reported having partners suffering from substance abuse that compromised the nurturing care environment through inconsistent caregiving, poor communication and emotional neglect. Other studies have reported similar findings and added that substance abuse might be a consequence of self-medicating to ease social isolation and stigma but has the damaging effect of contributing to children’s poor mental health outcomes including anxiety, depression and maladaptive coping strategies (28, 29). Evidence consistently shows that caregiver depression could also trigger the use of drugs to cope with stress and unsupportive or violent spouses. Furthermore, this could also increase the likelihood of neglect, harsh parenting, and poorer child outcomes (30), while accessible mental health support bolsters the capacity to provide nurturing care (31). Caregivers’ mental health, including issues related to addiction to drugs and alcoholism, should be addressed to ensure freedom from harm within families of children with disabilities and to prevent the deterioration of family functioning. Parenting programs have successfully been used and achieved a reduction in the trend of alcohol abuse and domestic violence among families affected by HIV in Rwanda (32). Besides, Interventions should also include components focusing on mental health support for caregivers to reduce harmful behaviors and improve the overall safety of the household (4). Offering community support systems and counseling for caregivers could help mitigate this risk.

Father’s abandonment was another significant issue raised by participants in our study population exposing the mothers to bear the disproportionate share of family obligations resulting in disrupted family functioning. Besides the mother’s loneliness, social isolation related to stigmatization due to the child’s disability is common among families of children with disabilities. Some studies in Africa have also documented father’s abandonment in families of children with disabilities (33, 34) while, father engagement was found to be beneficial to child development (35). In fact, the presence of both parents, during the antenatal care visit and in post-partum, is very crucial to maintain family cohesion. The pregnancy and its outcomes are usually unpredictable, and the perinatal period is a stressful moment for caregivers, especially new parents. Enrollment and retention in a parenting program alongside father engagement could be critical supports during this period. Systematic reviews conducted in Low-and middle-income countries noticed that parenting programs didn’t anticipate their adaptation to men and omitted taking into account violence prevention, gender dynamic and financial support (36). Other studies also showed that considering the cultural and societal context can improve father commitment (37). Fathers with alcohol abuse and low levels of education were found to be at risk of parenting program drop out (38).

Caregivers of children with disabilities in rural areas cited still struggling to access basic physiological, healthcare and child’s education. Our findings are similar to studies (39) (7) that identified different facets of health challenges such physical, emotional and social as main factors that interventions should target. The rural areas present an additional challenge in access of health care and education services because of remote location, financial constraints, negative beliefs related to child disabilities, insufficient trained professionals related to child disabilities, lack of transportation and inadequate policy implementation (40–42). Interventions should focus on improving access to basic needs, particularly for families of children with disabilities, and advocating financial coaching and support through government programs. Also, interventions play a key role in promoting school readiness by improving access to affordable learning and playing materials, coaching parents at home, particularly for families of children with disabilities (43). Support through government programs or subsidized adequate instruments for vulnerable families could ensure that additional needs are met. Interventions should foster family support systems that alleviate the burden on single mothers. This can include community-based caregiving initiatives that allow mothers to pursue economic opportunities while ensuring their children are cared for. ECD centers may emerge as a stress reliever. Again, despite progress, the ECD program remained insufficiently inclusive and failed to fully assist children with special needs (44). Therefore, there is a need for home visiting interventions that integrate ingredients that provide a more set of services for an optimal stimulation for unlocking the full potential of children with developmental disabilities (45).

At the social level, for family and connection with others, community education is important. Caregivers of children with disabilities reported feeling isolated and excluded from contributing to the development of their community. Our findings are similar to other studies that found that families and children with disabilities would like to be involved in the community. The studies noticed a gain in quality of life for caregivers and their children when they get access to community-based support systems that can enhance the sense of connection and shared responsibility for them (41, 46). Education and peer support groups and community sensitization programs can also combat stigma. Similar findings were noticed in different other studies identifying where stigmatization was linked to poor parental and community education and lack of social support. Some of the coping strategies were associated with a risk of compromising family functioning, increasing stress and leading to burnout (47). In our study caregivers stated that they were struggling with accessing livelihood due to lack of money to buy necessities, lack of time for acquiring self-skills and employment. Our findings are similar to other studies including a scoping review that noticed the access to social protection and self-employment as factors to take into consideration to improve caregiver’s livelihood and ease the family economic burden (23). Additionally, Linkages to cash transfers, livelihood support, Village Savings and Loan Associations (VSLAs), and subsidies for home modifications or assistive devices can strengthen family resilience if implemented alongside with supplemental non-economic intervention (48).

## 6 Policy recommendations for governmental or structural reforms

To improve family functioning among caregivers of children with disabilities, the government policies and stakeholders should gear its policies towards targeting the fathers at risk of family abandonment during the perinatal period. The structural reforms should emphasize the father’s role in childhood development as important along with screening caregiver’s wellbeing (49) and engagement in parenting practice. Promoting family reunification and involving fathers in disability education, and caregiving may help reduce abandonment. This can be achieved through counseling services and awareness programs to address cultural misconceptions (50).

In Rwanda, a parenting program tailored to cultural context called “Sugira Muryango” was able to increase father’s engagement through the creation of male champions, household home visitation, saving groups and credit association creation (51). The same parenting program was found to reduce home violence and promote father engagement (52). Support networks for single mothers, including emotional and social support, should be strengthened to ensure the emotional safety and protection of these families. Peer support groups, counselling, stress-management training, and routine screening for caregiver depression should create emotional scaffolding around families. Community dialogues, counselling that fosters positive attitudes, sibling sessions, and public messages celebrating abilities would help dismantle stigma. Stigma often leads to children being hidden, excluded from services, or denied early intervention opportunities (53). Inclusive attitudes and communities would create safer, more nurturing environments that protect child wellbeing (54).

Additionally, families of children with disabilities would benefit from services providing screening for feeding difficulties, guidance on adaptive positioning, and referral to nutrition rehabilitation to ensure rehabilitation ensures that children at heightened risk of malnutrition or growth faltering receive appropriate support (17, 55). Integrating disability-sensitive nutrition aligns with the nurturing care principles of supporting optimal physical development (45). Routine developmental screening integrated into ECD centers, coupled with strengthened CHW capacity, can facilitate early identification and timely referral. Evidence shows that early detection significantly improves developmental outcomes and reduces long-term risks (11, 56, 57). When properly trained, community health workers reliably identify developmental delays even in low-resource settings (58).

In rural Rwanda, leaders should make sure that every voice is heard during the Imihigo meetings. Interventions should advocate policy adjustments to expand access to health insurance for families with children with disabilities, particularly those in low-income households (59). Exploring micro-insurance schemes or community health initiatives might be beneficial. Providing healthcare access for caregivers themselves, ensuring that their well-being is maintained so they can continue to care for their children and improve their family functioning. Interventions should advocate for affordable healthcare options and targeted financial support to prevent families from spiraling into deeper poverty due to healthcare costs (60, 61).

A home visit program encouraging caregivers to participate in village saving loans (Ibibina) for vulnerable families could ensure that a starting point in life is achieved and a sustainable response to basic needs is met (51). Additionally, Programs that provide economic support to families, such as cash transfer programs or income-generating activities, should be integrated into interventions to ensure these families are financially secure while they care for their children (62). Initiatives should encourage community participation in sharing caregiving duties and providing resources, which can help mothers gain economic independence. This can also include vocational training programs and Village Savings and loans (Ibibina) initiatives aimed at empowering single mothers.

## 7 Limitation

Our study only interviewed a few fathers, which may have resulted in an unbalanced information related to father abandonment. It is possible that some fathers left the house for other reasons, including the other partner’s attitude. A second limitation was that we were not able to capture more profound and systemic causes of family dysfunction, including (historical, legal, political and socio-economic environmental factors), which was beyond the scope of this paper.

## 8 Conclusion

The SAFE framework provided a potential source of child protection’s ingredients that highlight the interconnection between safety, access to healthcare, family support, and economic security. Addressing these interconnected domains will enable a comprehensive approach to supporting families of children with disabilities in Rwanda, ensuring resilience despite adversity. This model can also guide the adaptation of interventions like Sugira Muryango to meet the specific needs of these families and improve their better functioning. Further studies are needed to understand the deep reason of father abandonment and father’s wellbeing.

## Data Availability

The original contributions presented in the study are included in the article/supplementary material. Further inquiries can be directed to the corresponding author.
